# Phylogenetic relationships, stage-specific expression and localisation of a unique family of inactive cysteine proteases in *Sarcoptes scabiei*

**DOI:** 10.1186/s13071-018-2862-0

**Published:** 2018-05-16

**Authors:** Deepani D. Fernando, Simone L. Reynolds, Martha Zakrzewski, Ehtesham Mofiz, Anthony T. Papenfuss, Deborah Holt, Katja Fischer

**Affiliations:** 1 QIMR Berghofer Medical Research Institute, Infectious Diseases Program, 300 Herston Road, Herston, Brisbane, QLD 4006 Australia; 2 School of Veterinary Sciences, University of Queensland, Gatton, QLD 4343 Australia; 3 Department of Veterinary Pathobiology, Faculty of Veterinary Medicine and Animal Science, University of Peradeniya, Peradeniya, Sri Lanka; 4 Bioinformatics Division, Walter and Eliza Hall Institute of Medical Research, Parkville, Victoria 3052 Australia; 5 Peter MacCallum Cancer Centre, Victorian Comprehensive Cancer Centre, Melbourne, 3000 Australia; 6 Department of Medical Biology, University of Melbourne, Parkville, Victoria 3010 Australia; 7 Menzies School of Health Research, Charles Darwin University, Casuarina, Northern Territory Australia

**Keywords:** *Sarcoptes scabiei*, Scabies, Mites, Skin Infection, Inactive proteases, SMIPP-Cs, Gene expression, Immunohistology, Phylogeny

## Abstract

**Background:**

Scabies is worldwide one of the most common, yet neglected, parasitic skin infections, affecting a wide range of mammals including humans. Limited treatment options and evidence of emerging mite resistance against the currently used drugs drive our research to explore new therapeutic candidates. Previously, we discovered a multicopy family of genes encoding cysteine proteases with their catalytic sites inactivated by mutation (SMIPP-Cs). This protein family is unique in parasitic scabies mites and is absent in related non-burrowing mites. We postulated that the SMIPP-Cs have evolved as an adaptation to the parasitic lifestyle of the scabies mite. To formulate testable hypotheses for their functions and to propose possible strategies for translational research we investigated whether the SMIPP-Cs are common to all scabies mite varieties and where within the mite body as well as when throughout the parasitic life-cycle they are expressed.

**Results:**

SMIPP-C sequences from human, pig and dog mites were analysed bioinformatically and the phylogenetic relationships between the SMIPP-C multi-copy gene families of human, pig and dog mites were established. Results suggest that amplification of the SMIPP-C genes occurred in a common ancestor and individual genes evolved independently in the different mite varieties. Recombinant human mite SMIPP-C proteins were produced and used for murine polyclonal antibody production. Immunohistology on skin sections from human patients localised the SMIPP-Cs in the mite gut and in mite faeces within in the epidermal skin burrows. SMIPP-C transcription into mRNA in different life stages was assessed in human and pig mites by reverse transcription followed by droplet digital PCR (ddPCR). High transcription levels of SMIPP-C genes were detected in the adult female life stage in comparison to all other life stages.

**Conclusions:**

The fact that the SMIPP-Cs are unique to three *Sarcoptes* varieties, present in all burrowing life stages and highly expressed in the digestive system of the infective adult female life stage may highlight an essential role in parasitism. As they are excreted from the gut in scybala they presumably are able to interact or interfere with host proteins present in the epidermis.

**Electronic supplementary material:**

The online version of this article (10.1186/s13071-018-2862-0) contains supplementary material, which is available to authorized users.

## Background

Scabies is a contagious disease caused by the obligatory parasitic burrowing mite *Sarcoptes scabiei.* This parasite can infect over 100 species of mammals, including humans [1]. The estimated number of human cases every year is between 100–300 million, which is around 2–3% of the world population [2]. Along with tinea and bacterial skin infections, scabies is one of the most common infectious skin disorders [3]. As scabies is highly contagious and transmitted through contact with infected skin or fomites it is predominantly seen in overcrowded living conditions, typically in economically disadvantaged populations [4]. Young children and the elderly are more commonly affected [5]. Importantly, in tropical climates the initial infection by mites facilitates the invasion of the affected skin with opportunistic, potentially pathogenic bacteria, particularly *Streptococcus pyogenes* and *Staphylococcus aureus*. The potential resultant complications include pyoderma, cellulitis, lymphangitis, sepsis, acute post-streptococcal glomerulonephritis, rheumatic fever and rheumatic heart disease [6].

There is no vaccine for scabies, and the few available broad-spectrum anti-parasitic drugs currently used often fail to control the disease [4, 5, 7, 8]. For example, the relatively short half-life of ivermectin in the skin [9] dictates repeated consecutive treatments, which causes considerable compliance and management issues. Emerging mite resistance against this leading drug is also of growing concern [10, 11]. Therefore, new therapeutic options are required. Recent research outputs of scabies mite EST libraries [12], genome [13, 14], proteome [15, 16], transcriptome (manuscript in preparation), established RNAi [17] and re-purposing of FDA approved drugs [9] will accelerate the finding of new therapeutics for this worldwide problem.

Parasite-encoded proteases are essential for regulatory interactions between parasites and their hosts, and thus are considered attractive anti-parasitic drug and/or vaccine targets [18]. Parasitic cysteine proteases are known for their roles in digestion [19], immune evasion [18, 20, 21], enzyme activation in host tissues [22], virulence [23], tissue and cellular invasion [24, 25], excystment [26], and hatching and moulting [24, 27]. Studies emphasised the immune evasion role of parasitic cysteine proteases in evading, suppressing and subverting the host immune responses [28, 29]. Due to their essential roles in the parasitic lifestyles these proteins have been considered as vaccine candidates against many parasites including ectoparasites [30, 31], and cysteine protease inhibitors have shown promising results in the control of some parasitic diseases [32, 33].

Scabies mites feed when burrowing within the epidermis [34] and ingest a multitude of diverse host proteins. The feeding success of scabies mites depends on their ability to digest epidermal and plasma components with proteolytic enzymes and to locally modulate the host complement and coagulation systems by releasing pharmacologically active proteins. In the past decade considerable data has been generated indicating that scabies mites express excretory gut proteins involved in these roles [35–37], including one serine protease [38] as well as multiple proteolytically inactive serine protease paralogs (SMIPP-Ss) [39–41] and serine protease inhibitors (Scabies Mite Serpins, SMSs) [42], both with novel host complement-inhibitory functions. There are likely to be multiple specific adaptations within each protein class produced by the mite that are important in the parasite’s life-cycle. Exploring these functions may enable us to design specific strategies to interfere with the mite survival.

With this overarching strategy in mind we aimed here to generate fundamental data of a previously discovered unique class of scabies mite cysteine proteases. These are amplified within the scabies mite genome into a multicopy gene family comprised of five proteolytically active and five predicted inactive members featuring mutations in their catalytic sites [43]. The protein products of these genes were termed Scabies Mite Inactivated Cysteine Protease Paralogs (SMIPP-Cs). The closest homologs to the *S. scabiei* cysteine proteases are the group 1 allergens of house dust mite (HDM), which are proteolytic papain-like cysteine proteases that can induce the pathogenic process of asthma and allergy [44–46].

Remarkably, in contrast to the expansion within the scabies mite genome, only a single gene encoding the group 1 cysteine protease allergen has been identified in the close relatives of scabies mites, namely *Der p* 1, *Der f* 1 and *Eur m* 1 in the free living HDM species *Dermatophagoides pteronyssinus*, *D. farinae* and *Euroglyphus maynei*, and *Pso o* 1 in the non-burrowing sheep scab mite *Psoroptes ovis*.

The five Scabies Mite Inactive Cysteine Proteases (SMIPP-C a-e), are not only distinct from other parasitic cysteine proteases, they are also distinct from their five active counterparts (*Sar s* 1 a-e). In each SMIPP-C the active cysteine has been replaced by a serine. This may or may not lead to inactivation of proteolytic properties of the proteases. In addition, in two of the SMIPP-Cs the active histidine has been replaced by a glutamine (SMIPP-Ca and SMIPP-Cb) and the active histidine of the three other SMIPP-Cs (SMIPP-Cc, SMIPP-Cd and SMIPP-Ce) has been replaced by a leucine. In addition, a glutamine at position 34 of three SMIPP-C sequences (SMIPP-Cc, SMIPP-Cd and SMIPP-Ce) has been replaced by glutamic acid, which has the potential to disturb the formation of the oxyanion hole during hydrolysis [43]. Hence, it has been proposed that these proteases are not able to form a thiolate-imidazolium charge relay diad, and are proteolytically inactive.

Proteolytically inactive proteases, with changes in their catalytic residues or with steric or structural rearrangements obscuring the active site and substrate binding pocket, have been shown to accomplish remarkable functions in biological processes including regulation, inhibition and immunomodulation [47, 48]. The SMIPP-Cs have not been reported in free-living HDMs and were not observed among HDM expressed sequence tags [43]. Consequently, it has been proposed that the presence of the SMIPP-S and SMIPP-C families in burrowing, parasitic scabies mites may be an adaptation to parasitism [39, 43]. Unlike free-living mites, *S. scabiei* is in direct contact with and must evade host defence mechanisms. If they have essential roles in this context, the SMIPP-Cs may be target proteins for novel immune or chemotherapeutic intervention strategies against scabies.

To elucidate their key functions and to determine if SMIPP-Cs are a potential target to control scabies infection, we have addressed here a range of mandatory key questions regarding their representation and phylogeny across a range of host-specific *S. scabiei* varieties, their precise location within the mite and in the infected host epidermis and their transcriptional levels in the successive stages of the mite life-cycle.

## Methods

### Sequence alignment and phylogenetic analysis of SMIPP-Cs

Complete amino acid sequences of HDM group 1 allergens; *Der p* 1, *Der f* 1 and *Eur m* 1, *S. scabiei* var. *hominis* active cysteine proteases *Sar s 1* a-e and *S. scabiei* var. *hominis* SMIPP-C a-e (accession numbers in Additional file 1: Table S1) were aligned using CLUSTAL O (1.2.0) [49]. Signal sequence and the pro-peptide for individual sequences were predicted using SignalP 4.1 [50] and SMART [51] servers. Glycosylation sites and disulfide bonds were predicted using NetNGlyc 1.0 [52] and the DiANNA 1.1 web server [53], respectively. *In silico* analyses using MULTALIN [54] and EMBOSS [55] were performed to understand sequence identity among SMIPP-Cs and with the active scabies mite cysteine proteases. SMIPP-C protein sequences from human [12] and dog [14] scabies mites (Additional file 1: Table S1) were used as queries for a local NCBI tBLASTn search to identify homologues of SMIPP-C sequences in the *S. scabiei* var. *suis* genome (pig_unwashed and pig_washed3) [13] (Additional file 1: Table S1) using an E value threshold of 10^-5^. Contig sequences were translated to their amino acid sequences and aligned with protein sequences of *Sarcoptes scabiei* type *hominis Sar s 1* allergen (Yv4003H01, Yv9053H09, Yv6030H07) using MAFFT [56]. TrimAl software was applied to remove poorly aligned regions [57]. Prottest v3.4.2 was used to determine the evolutionary model that best fit the data [58], which corresponded to WAG + G + I. A Bayesian phylogenetic tree was calculated using MrBayes software package v3.2.7 with 100,000 generations and sampling every 100 generations [59]. Bayesian posterior probabilities were determined after the initial ‘burn-in’ period corresponding to 12.5% of the generations. A maximum likelihood tree was generated using RAML v8.2.11 [60]. Bootstrapping values were calculated based on 100 iterations.

### Sequence analysis, cloning, expression and purification of SMIPP-C proteins

Five genes from the *S. scabiei* var. *hominis* SMIPP-C family have previously been identified [43]. Three SMIPP-Cs from three different clades of the SMIPP-C protein family, SMIPP-Ca (cDNA clone Yv4025A02), SMIPP-Cc (cDNA clone Yv5009F04) and SMIPP-Ce (cDNA clone Yv4028C12) were selected for this study (Additional file 1: Table S1). Predicted mature protein sequences were obtained from NCBI [12] and primers were designed with restriction sites *BamH*I and *Not*I for SMIPP-Ca and *Sal*I and *Pst*I for SMIPP-Cc and SMIPP-Ce to achieve directional cloning into the pQE-9 expression vector (Qiagen, Hilden, Germany), in frame with N terminal 6× Histidines tags of the expressed proteins. SMIPP-Ca forward primer (5′-acc ggg atc cCA AGA ATT GAC TGA ATC TCC TCC G-3′), SMIPP-Ca reverse primer (5′-acc gct gca gtt aGA ATT CAG GTC GAC CCA ATC TGA C-3′), SMIPP-Cc forward primer (5′-acc ggt cga cTA TTA TTT CGA GAC AAC GCC AAG TGA TGC TG-3′) SMIPP-Cc reverse primer (5′-acc gct gca gtc aTT CAA AAT CTT CAG GCT CAT TTT CAA AAG G-3′), SMIPP-Ce forward primer (5′-acc ggt cga cTA TTA TTT TGA GAC AAC GCC TAG TAT TG-3′) and SMIPP-Ce reverse primer (5′-acc gct gca gtc aGG AAT CAT CGG GCT CAG CTT CAA AAG G-3′) were synthesised by Sigma-Aldrich, Australia. Lower case, underlined sections of the primer sequences indicate the incorporated restriction sites. PCR amplified sequences were digested at their restriction sites and ligated into the linearised pQE-9 vector. Ligated vector was transformed into XL1 blue *Escherichia coli* competent cells and selected on Luria broth (LB)/ampicillin (100 μg/ml) agar. Transformants were confirmed by BigDye 3.1 (Applied Biosystems, Foster City, CA, USA) sequencing using pQE-9 sequence-specific primers.

Sequence confirmed clones were transformed into BL21 *E. coli* competent cells and proteins were expressed. Briefly a single colony of BL21 *E. coli* cells was cultured in LB medium containing 100 μg/ml ampicillin at 37 °C with 230× *rpm* orbital shaking up to OD_600_ value 0.5–0.6. Protein expression was induced by the addition of Isopropyl β-D-1-thiogalactopyranoside (IPTG) to a final concentration of 1 mM for 4 h. The culture was centrifuged at 6000× *g* for 20 min at 4 °C, and the cell pellet was resuspended in 15 ml of lysis buffer (50 mM Tris, 10 mM EDTA, 100 mM NaCl, pH 8.0) containing 1 mg/ml lysozyme, 10 μg/ml DNase, 5 μg/ml RNase, 0.4 ml/10 ml cOmplete™ EDTA-free protease cocktail (Sigma-Aldrich, St. Louis, MO, USA) and 2 mM MgCl_2,_ and incubated for 1 h at room temperature (RT) on a roller. The suspension was homogenised several times using a Potter-Elvehjem homogeniser and sonication of spheroplasts was done by Sonifier 250 (Branson, USA) for 6 times of 30 sec burst cycles with 30 s cooling intervals in between on ice. The cell lysate was centrifuged at 12,000× *g* for 10 min at 4 °C and the proteins were detected in the inclusion body cell pellet by Coomassie blue stained 10% SDS PAGE. Inclusion bodies were washed 6 times with 10 ml of lysis buffer containing 0.5% (v/v) Triton-X 100 and recovered by centrifugation (16,000× *g*, for 20 min at 4 °C). The washed inclusion bodies were solubilised in 4 ml of 6 M guanidine hydrochloride, 50 mM Tris, pH 8.0, 100 mM NaH_2_PO_4_.H_2_O and 1 mM DTT and bound overnight onto an immobilised and pre-equilibrated Ni-NTA matrix (Qiagen) at 4 °C with circular rotation. Unbound protein flowthrough was collected by gravity flow and the column was washed twice with 5 mM and 10 mM imidazole wash buffers (6M urea, 100 mM NaH_2_PO_4_.H_2_O, 10 mM Tri-NaOH, 150 mM NaCl pH 8.0, 1% (v/v) glycerol and 1 mM DTT) to remove non-specifically bound proteins. Target proteins were eluted with 250 mM imidazole in wash buffer at pH 8.0. The purity was confirmed and proteins were quantified relative to a series of bovine serum albumin (BSA) standards by SDS PAGE analysis with Coomassie blue staining.

### Antibody preparation

Six BALB/c female mice per protein were used for antibody production by immunisation with purified recombinant SMIPP-C proteins. Pre-immune sera were collected from the mice before the protein injections and pooled. Acetone-precipitated SMIPP-C recombinant proteins were resuspended in 1× PBS buffer and used to immunise the mice. Antibody production was initiated by subcutaneous injection of 50 μg recombinant protein emulsified in Freund’s Complete adjuvant (Sigma-Aldrich, USA) and boosted twice with 2 weeks interval with the same amount of protein doses emulsified with Freund’s Incomplete adjuvant (Sigma-Aldrich, USA). After the third immunisation, antibody production and specificity were tested using Odyssey® western blot. Odyssey® western blot in brief: SMIPP-C proteins were loaded separately on 10% SDS PAGE and transferred to an Immubolin-FL PVDF membrane (Merck Millipore, Temecula, CA, USA). All following incubation steps were done at RT for 1 h with gentle orbital shaking unless stated otherwise. The membrane was blocked with Odyssey® blocking buffer (LI-COR Biosciences, Lincoln, NE, USA) followed by incubation with 1:500 diluted mouse sera and washed 3 times with PBS-0.05% TWEEN® 20 (PBST). Bound antibodies were detected by incubation with goat anti-mouse-IR 800 nm secondary antibodies (LI-COR Biosciences) 1:10,000 and the membranes were washed 3 times as before. Antibody binding was visualised by the Odyssey Infrared Imaging System (LI-COR Biosciences, USA) and images were analysed using the Image Studio™ Lite 5.2.5 software. Mouse serum was harvested upon positive results. Antibody cross reactivity was tested between each SMIPP-C protein and against active cysteine protease *Sar s* 1 using Odyssey® western blot as described above.

### Immuno-histological localisation

The localisation of SMIPP-C proteins within and in the vicinity of the scabies mite was demonstrated using sections of human skin infested with mites probed with the polyclonal antibodies raised in mice against individual SMIPP-Cs. Adjacent serial sections were probed with anti-human IgG, which is known to be ingested by the mite [61] and therefore serves as a marker to localise gut tissue. Adjacent serial sections were also probed with pre-immune mouse serum as a negative control. Paraffin blocks of 5 mm^3^ scabies-infected human skin tissues [62] were used to cut 4 μm sections and coated on X-Tra™ (Leica Biosystems, Nußloch, Germany) glass slides. The slides were dried at 37 °C for 3 h and dewaxed in xylene followed by graded ethanol. All the incubation steps were done at RT in a humidifier chamber and all the washes were 3 times of 5 min with Tris Buffered Saline (TBS pH 7.6) unless stated otherwise. Endogenous peroxidase activity was blocked with 3% H_2_O_2_ in TBS for 10 min and the slides were washed. Non-specific protein binding was blocked with 10% goat serum in TBS for 30 min. Excess serum was decanted and the slides were probed with pre-immune mouse sera (negative control) or with SMIPP-C specific antibody (test) overnight at 4 °C. Test and negative control slides were washed and probed with anti-mouse probe MACH1 (Biocare Medical, Pacheco, CA, USA) secondary antibody for 20 min at RT. Both slides were first washed with TBST followed by 2 washes with TBS and probed with MACH1 universal HRP polymer (Biocare Medical) for 20 min followed by 3 TBST washes. A third serial tissue section (positive control), blocked for endogenous peroxidase activity and nonspecific binding, was probed with horseradish peroxidase (HRP) labelled anti-human IgG (Sigma-Aldrich, USA) for 1 h at RT as a mite gut marker [61]. It was followed by a wash with TBST and 2 subsequent washes with TBS. Nova-RED substrate (VECTOR LABORATORIES, Burlingame, CA, USA) was added to all three sections to initiate the chromogenic reaction and it was stopped by immersion in deionised water for 3 min at RT. The slides were counterstained with haematoxylin for 1 min, dehydrated in graded ethanol, cleared in xylene and mounted with DPX histology slide mounting medium (Sigma-Aldrich, USA). Slides were visualised using an Aperio XT Scanscope (Leica Biosystems) slide scanner at 40× magnification and analysed using eSlide manager and ImageScope viewing softwares (Leica Biosystems).

### Identification of *S. scabiei* var. *hominis* life stages

Between 20–100 individual organisms representing the different life stages of *S. scabiei* var. *hominis* preserved in TRIzol (Invitrogen, Carlsbad, CA, USA) were separated first under a stereo binocular dissecting microscope and subsequently males and nymphs were further separated under a bright field microscope at 40× magnification. Eggs are glossy, whitish and ovoid with enclosed embryo or larvae in its foetal stage. Larvae have only 3 pairs of legs and are similar in size to the eggs. Females are the largest and have an ovoid creamy-white coloured body with 4 pairs of legs; 2 pairs of anterior legs end with small suckers and the hind 2 pairs end with long seta. Males are approximately two thirds the size of a female and similar in size to nymphs. Males and nymphs have 4 pairs of legs; however, a distinctive difference under the bright field microscope (at 40× magnification) is that both pair of hind legs end with long seta in nymphs, but in males the inner pair of hind legs terminates as a broad pad [63].

### Amplification of SMIPP-C transcripts by ddPCR

SMIPP-Ca, SMIPP-Cc and SMIPP-Ce primers were designed to amplify relatively conserved regions containing 123 bp, 151 bp and 98 bp, respectively for droplet digital PCR (ddPCR). Primer sequences are, SMIPP-Ca forward primer (5′-GCG AGG AAA ATG TCA AGA GA-3′), SMIPP-Ca reverse primer (5′-GGG CAC CGT ATG CGG ACA AAT-3′), SMIPP-Cc forward primer (5′-CAG ACA GGC GCG ATT AGA AC-3′), SMIPP-Cc reverse primer (5′-CCA CGA TGC TTT ACA TTT ACT TTC GGT TG-3′), SMIPP-Ce forward primer (5′-CCG AGA TTA TTG GGT CGT TAA G-3′), SMIPP-Ce reverse primer (5′-CCA AGA TAC CGA AAA GAT TCT CTT CC-3′). The primers were used to amplify the SMIPP-C fragments from *S. scabiei* var. *hominis* cDNA libraries [12] by conventional PCR with cycling conditions; 95 °C for 10 min, 35 cycles of 95 °C for 30 s, 54 °C for 30 s and 72 °C for 1 min, and final extension at 72 °C for 7 min. DNA negative and primer negative controls were also included. Resulting products were cloned into the pUC19 vector using a PCR cloning kit (NEB, Ipswich, MA, USA) according to the manufacturer’s instructions. The primer specificity was confirmed by BigDye®3.1 sequencing (Applied Biosystems).

The sensitivity of the primer pairs was evaluated by testing different primer concentrations from 50 nM to 200 nM final concentrations; 100 nM final was the optimal concentration for all three sets of ddPCR primers in the reaction mix. Temperature gradient ddPCR conditions established the optimised annealing temperatures for the SMIPP-Ca, SMIPP-Cc and SMIPP-Ce ddPCR primers as 63 °C, 60 °C and 56 °C, respectively.

To investigate whether SMIPP-C transcription is stage-specific, different life stages of *S. scabiei* var. *hominis* mites preserved in TRIzol were separated, using previously established methods [63] under stereoscopic and bright field microscopes. *S. scabiei* var. *hominis* eggs, larvae, nymphs, males and females were collected separately into Direct-zol™ (Zymo Research, Irvine, CA, USA) and the Direct-zol™ RNA MicroPrep kit (Zymo Research) was used to extract mite total RNA according to the manufacturer’s instructions. cDNA was synthesised using Superscript® II reverse transcriptase (Thermo Fisher Scientific) and Oligo(dT)_20_ primer (Thermo Fisher Scientific). Prepared cDNA was used to amplify SMIPP-C transcripts by ddPCR using specific primers. Reactions were performed in 20 μl reactions, consisting of 1× EvaGreen supermix (Bio-Rad, Hercules, CA, USA), 100 nM forward primer, 100 nM reverse primer, 1 μl cDNA and molecular grade water (Invitrogen) in a semi-skirted twin.tec 96 well PCR plate (Eppendorf AG, Hamburg, Germany). ddPCR cycling times were 95 °C for 10 min followed by 45 cycles of 95 °C for 30 s, annealing temperature for 30 s and 72 °C for 1 min, and final extension at 72 °C for 10 min followed by a single final dye stabilization step of 4 °C for 5 min and 95 °C for 5 min. SMIPP-C gene transcription was quantified using a QX200 droplet digital PCR system (Bio-Rad) and analysed using the appropriate automated QuantaSoft software (Bio-Rad). Target gene transcription was calculated as SMIPP-C gene transcript copy numbers per individual mite or egg. All error bars were generated by QuantaSoft software and represent a 95% confidence interval.

## Results

### Sequence alignment of the *S. scabiei* var. *hominis* SMIPP-C family

**Fig. 1:**
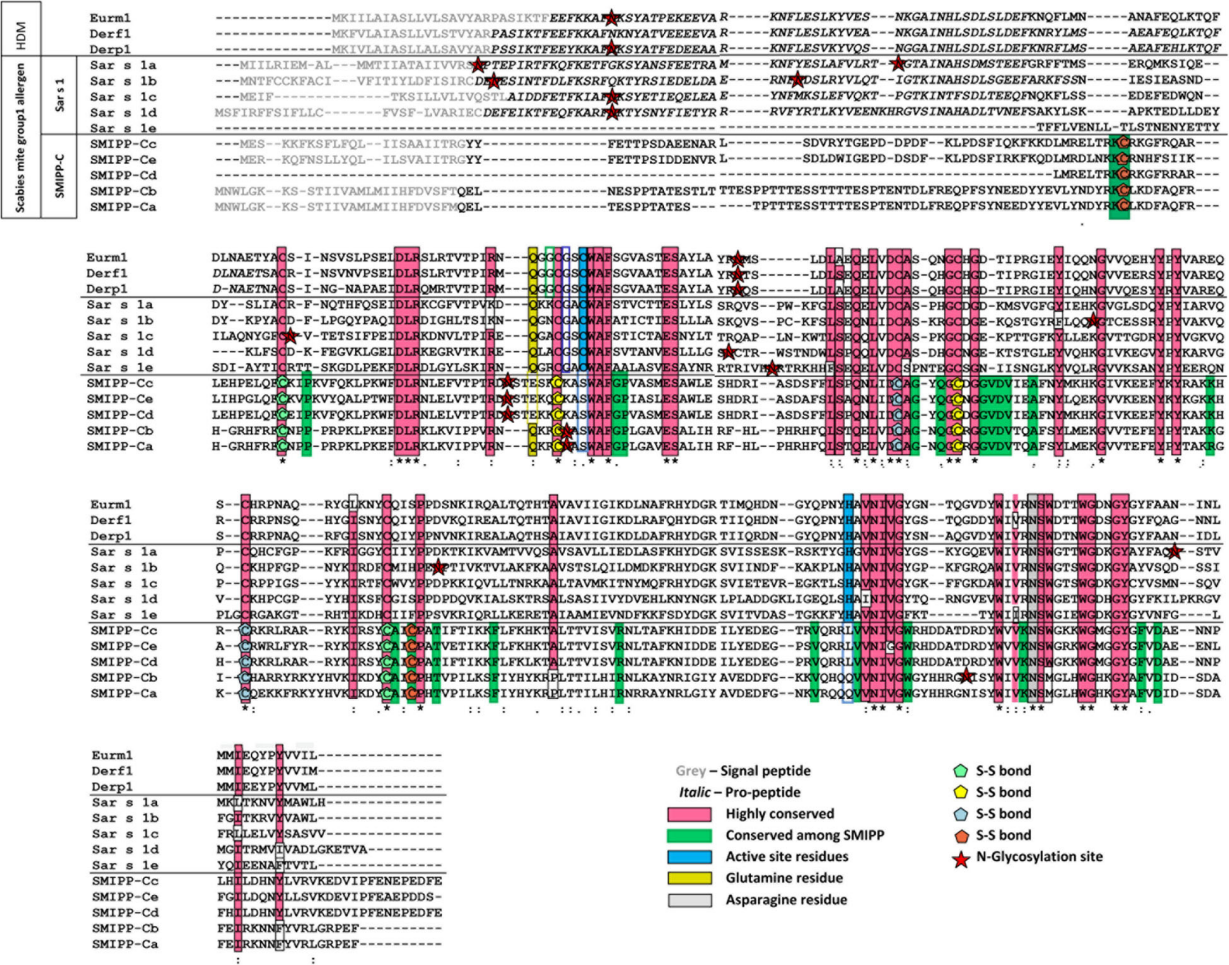
*In silico* analysis of HDM group 1 allergens, *Sar s* 1 a-e and SMIPP-C a-e. CLUSTAL O (1.2.0) alignment of the protein sequences of HDM group 1 allergens and the scabies mite homologs *Sar* s 1 a-e and SMIPP-C a-e. The signal sequences are printed in grey and the pro-peptide regions in italic. Conserved residues and the residues important for catalytic activity and specificity are highlighted in colour as indicated. Predicted disulfide bonds and glycosylation sites are indicated by pentagon and star symbols, respectively

Homologous features and differences between the group 1 HDM allergens the active SM cysteine proteases *Sar s* 1 a-e and the SMIPP-Cs are shown in the CLUSTAL O  alignment of the protein sequences in Fig. 1. Eight out of ten scabies mite proteins have a confirmed signal sequence, indicating their secretory nature, as is seen with the HDM group 1 allergens. All SMIPP-Cs however lack a predicted pro-peptide and have further distinct features. While they share regions of homologous sequence with the active protease sequences (highlighted in pink in Fig. 1), they display unique conserved regions which could potentially result in different structural and functional properties (Fig. 1, green highlighting). The mutated catalytic sites and mutations in the glutamine, which is important for oxyanion hole formation, are indicated in blue and yellow, respectively [43]. All five SMIPP-Cs have four predicted disulfide bonds (Fig. 1, pentagons) compared to between 3 and 7 disulfide bonds predicted in the proteolytically active counterparts of HDMs and scabies mites (not shown). The pattern of predicted glycosylation is distinctively shifted in the SMIPP-Cs (Fig. 1, red stars). All of these differences between the SMIPP-Cs and their active equivalents may be noteworthy, as the distinct changes in pro-peptides, catalytic sites, disulfide bonds and glycosylation previously reported for the SMIPP-Ss [41] are thought to have led to structural changes and ultimately to an altered function [54, 55]. *Sarcoptes scabiei* var. *hominis* SMIPP-Cs have 22–26% protein sequence identity with the HDM group 1 allergen (*Der p* 1) and 21.5–27% protein sequence identity with the scabies mite active cysteine proteases (*Sar s* 1 a-e). The five members of SMIPP-C family have between 31–93% amino acid identity to each other. SMIPP-Ca and SMIPP-Cb as well as SMIPP-Cd and SMIPP-Ce show a very high protein identity of 93% and 81.5%, respectively.

### Phylogenetic analysis of human, pig and dog mite SMIPP-Cs

**Fig. 2:**
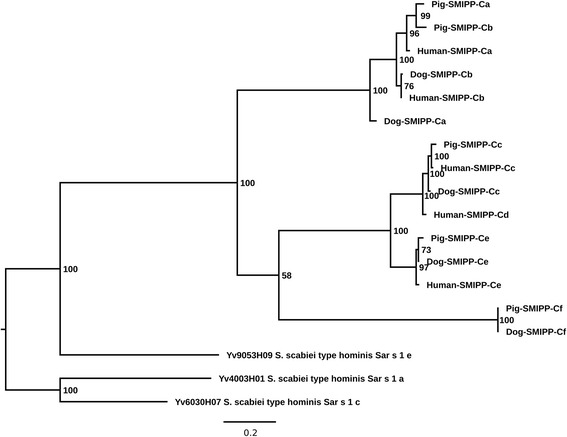
Phylogenetic relationships of SMIPP-Cs. The phylogenetic relationship between amino acid sequences of *S. scabiei* var. *hominis* (Human-SMIPP-C), *S. scabiei* var. *suis* (Pig-SMIPP-C) and *S. scabiei* var. *canis* (Dog-SMIPP-C) was estimated by Bayesian inference (accession numbers are provided in Additional file 1: Table S1). Numbers at nodes represent Bayesian posterior probabilities. The tree was rooted using *Sarcoptes scabiei* type hominis *Sar s *1 allergen protein sequences (Yv4003H01, Yv9053H09, Yv6030H07)

Five additional SMIPP-C sequences identified in the recently established *S. scabiei* draft genome databases of dog [14] and pig [13] mites were converted into amino acid sequences and were included in the phylogenetic analysis implemented using a Bayesian inference to complement previous phylogenetic tree analyses of SMIPP-Cs in human and dog mites [16, 43]. Phylogenetic comparison of SMIPP-Cs of *S. scabiei* var. *hominis* (human mite), *S. scabiei* var. *suis* (pig mite) and *S. scabiei* var. *canis* (dog mite) indicated that amplification of the SMIPP-C genes into a family of five occurred in a common ancestor and individual genes evolved independently in the different mite varieties (Fig. 2). Notably, the SMIPP-Cf lineage only occurred in pig and dog mites but not in human mites. The SMIPP-Cd is another variant of SMIPP-Cc and only occurred in human mites. The dog SMIPP-Ca is remarkably different compared to all other SMIPP-C variants. The results were confirmed in a phylogenetic analysis using a maximum likelihood approach implemented in RAxML (Additional file 2: Figure S1).

### Cloning, expression and purification of SMIPP-C proteins

**Fig. 3:**
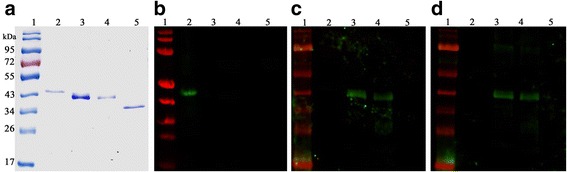
Specificity of mouse sera against recombinantly expressed and affinity purified SMIPP-Ca, SMIPP-Cc and SMIPP-Ce. Coomassie blue R-250 stained SDS PAGE (**a**) and western blots using antibodies against SMIPP-Ca (**b)**, SMIPP-Cc (**c)** and SMIPP-Ce (**d**) recombinant proteins. Lane 1: protein marker; Lane 2: SMIPP-Ca; Lane 3: SMIPP-Cc; Lane 4: SMIPP-Ce; Lane 5: *Sar s* 1c recombinant proteins. Predicted molecular weights of SMIPP-Ca, SMIPP-Cc, SMIPP-Ce and *Sar s* 1c are 36.16 kDa, 37.24 kDa, 36.46 kDa and 34.73 kDa, respectively

*Sarcoptes scabiei* var. *hominis* SMIPP-Cs from three different clades (SMIPP-Ca, SMIPP-Cc and SMIPP-Ce) were produced as His-tagged recombinant proteins. Fragments equivalent to the predicted mature amino acid sequences were amplified by PCR from existing expressed sequence tag (EST) libraries. Sequencing confirmed that SMIPP-Ca, SMIPP-Cc and SMIPP-Ce clones were 100% identical to the published GenBank sequences [12]. Mature sequence predicted molecular weights and isoelectric points including the C terminal His-tag were predicted to be 36.16 kDa and pI 9.24 for SMIPP-Ca, 37.24 kDa and pI 8.74 for SMIPP-Cc and 39.14 kDa and pI 6.13 for SMIPP-Ce. The proteins were expressed in BL21 *E. coli* cells, isolated from inclusion bodies under denaturing conditions and purified via affinity chromatography. Purified proteins from all 3 SMIPP-Cs were of high purity, as shown in a Coomassie blue stained gel analysis (Fig. 3a).

We immunised mice with purified recombinant SMIPP-C proteins and tested the antibody specificity by Odyssey® western blot. The polyclonal antibodies raised against SMIPP-Ca were highly specific and did not cross react with any other SMIPP-Cs tested (Fig. 3b; Lanes 2–4). The antibodies raised against SMIPP-Cc and SMIPP-Ce cross reacted with both SMIPP-Cc and SMIPP-Ce proteins (Fig. 3c, d; Lanes 3 and 4), but not with SMIPP-Ca (Fig. 3c, d; Lanes 2). Notably, none of the SMIPP-C antibodies cross reacted with their proteolytically active counterpart, the scabies mite active cysteine protease *Sar s* 1c (Fig. 3b, c, d; Lanes 5). A western analysis probing scabies mite extracts with the individual sera showed single bands of similar size for the three sera, indicating reactivity to the native proteins in the extract (Additional file 3: Figure S2). It was concluded that the antibodies raised against recombinant proteins of *S. scabiei* var. *hominis* SMIPP-Cs were suitable reagents for immunolocalisation of SMIPP-Cs expressed by scabies mites.

### Immuno-histological localisation of SMIPP-Cs

**Fig. 4:**
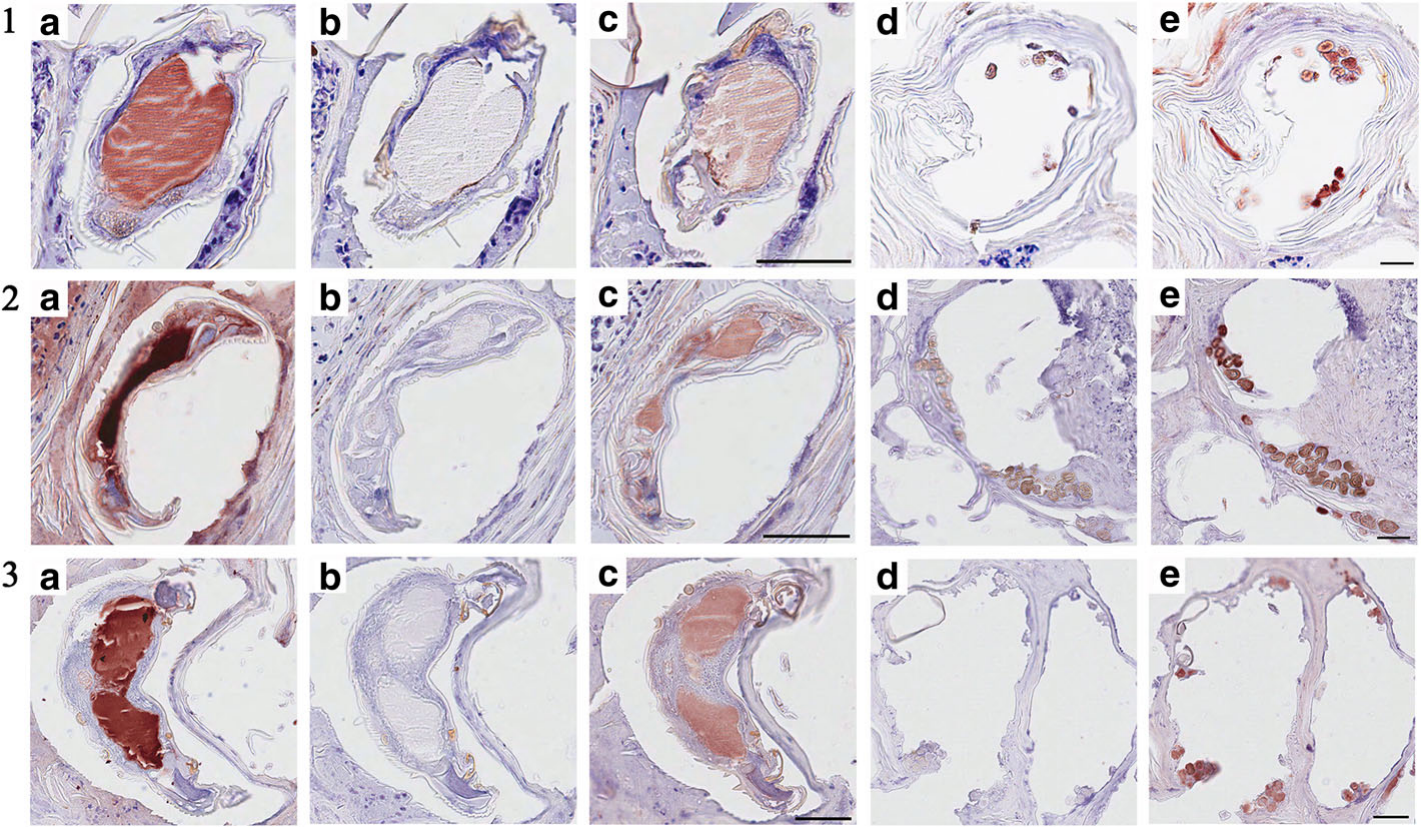
Localisation of SMIPP-Cs in *S. scabiei* var. *hominis* infected epidermal tissue by immuno-histochemistry. Series 1, 2 and 3 are serial histological sections of scabies mite infested human skin showing localised SMIPP-Ca, SMIPP-Cc and SMIPP-Ce proteins. **a-c** *S. scabiei* var. *hominis* mites within human skin burrows. **d**, **e** Mite faecal pellets. **a** Probed with anti-human IgG (mite gut marker). **b**, **d** Probed with pre-immune mouse sera (negative control). **c**, **e** Probed with SMIPP-C specific antibodies. Red staining indicates antibody binding to protein. *Scale*-*bars*: 50 μm

The localisation of SMIPP-C proteins within and in the vicinity of scabies mites in human skin was demonstrated using the polyclonal antibodies raised in mice against individual SMIPP-C proteins [61]. All three SMIPP-Cs investigated were localised to the digestive system of the mite (Fig. 4c). Sections probed with antibodies against SMIPP-Ca, SMIPP-Cc and SMIPP-Ce stained positive (red staining: Fig. 4, sections 1c, 2c and 3c, respectively) in regions that were identified as mite gut tissue with the gut-specific anti human IgG antibody (Fig. 4: sections 1a, 2a and 3a, respectively) and in mite faeces (Fig. 4e). Given the cross-reactivity of antibodies against SMIPP-Cc and SMIPP-Ce, immuno-histology using these probes may show localisation of SMIPP-Cc and/or SMIPP-Ce to the mite digestive system and mite faeces. All sections probed with pre-immune mouse sera (negative control) showed the counter stain and the unstained pale to dark brown colour of chitin and faeces (Fig. 4: sections 1b and d, 2b and d, and 3b and d).

### Amplification of SMIPP-C transcripts by ddPCR to evaluate the stage-specific SMIPP-C expression at the transcriptional level

**Fig. 5:**
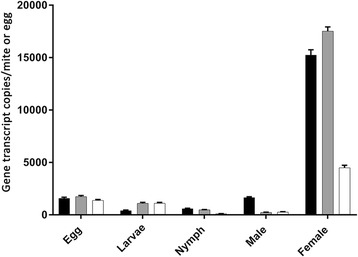
SMIPP-C expression profiles throughout the *S. scabiei* life-cycle. Transcriptional level gene expression of SMIPP-Ca (black), SMIPP-Cc (grey) and SMIPP-Ce (white) in *S. scabiei* var. *hominis*, quantified by Droplet Digital PCR (ddPCR). Error bars represent a 95% confidence interval

Initial primer testing and optimization of the PCR assays for the amplification of the selected three SMIPP-C sequences was done by conventional PCR using DNA extracted from pUC19 clones of SMIPP-Ca, SMIPP-Cc and SMIPP-Ce. No cross-amplification was observed, indicating that the SMIPP-C ddPCR primers were sequence-specific [63]. Between 20 and 100 single organisms representing each life stage (eggs, larvae, nymphs, adult males and adult females) were pooled. Stage-specific cDNA preparations were subjected to the established ddPCR assay. Using this methodology, we were able to quantify stage-specific SMIPP-C gene transcription and to calculate the gene transcript copy numbers per individual life stage. Compared to all other life stages, SMIPP-Ca, SMIPP-Cc and SMIPP-Ce were highly expressed in adult females (between 43 and 170 times higher), accounting for 15,259.3 ± 493.8, 17,530.9 ± 395.1 and 4508.6 ± 222.2 gene transcript copies per single female mite, respectively (Fig. 5).

## Discussion

The enzymatically inactive SMIPP-Cs described here are unique to parasitic scabies mites and have not been reported to be present in the closely related free living HDMs. We postulated previously that the evolution of SMIPP-Cs may be an adaptation to the parasitic state [39]. Since then SMIPP-Cs have not been observed among HDM ESTs [39] or in the more recent HDM genome database [64]. The proposed essentiality of the SMIPP-Cs for the parasitic scabies mite is further emphasised by their demonstrated presence in three *S. scabiei* varieties of medical and veterinary importance. The SMIPP-C proteins are homologous to the five scabies mite active cysteine proteases (*Sar s* 1 a-e) and to the group one allergen of HDMs [43]. Figure 1 illustrates that the SMIPP-C amino acid sequences are conserved among each other and distinctly different to the active cysteine proteases. Their unique sequence features are likely have distinct structural and functional properties. Our phylogentic analysis aligns with previous studies [15, 43], indicating that they are a monophyletic gene family. We propose that the SMIPP-Cs were amplified in the most common recent ancestor of the three mite varieties, and that the sequence variation between the mite varieties is consistent with sequence variation between the mite varieties at other loci. Within each scabies mite variety the SMIPP-Cs evolved into slightly different variants, presumably as the sub-speciation along with the adaptation to different hosts occurred. While the SMIPP-Cf lineage only occurred in pig and dog mites but not in human mites it seems that the human mites have two different versions of SMIPP-Cc and no SMIPP-Cf. The data indicate that the amplification happened prior to host species adaptation and that the sequence variation happened with host adaptation. We hypothesize that, provided the SMIPP-C proteins have an essential function for the parasite, these seemingly inconsequential differences in SMIPP-C evolution could contribute to the strict host specificity seen in scabies mites.

The fact that SMIPP-Cs are expressed and have been amplified into multicopy families indicates that despite their proteolytic inactivity, the SMIPP-C genes are not redundant pseudogenes. Despite their catalytic mutations and the expected lack of proteolytic activity in the encoded proteins, they may have evolved new functions and may interact with host proteins in different, non-proteolytic ways. A recent review summarised the extensive and expanding role of pseudoproteases in other regulatory functions and immune evasion using alternative binding sites or exosites [48]. For example, the proteolytically inactive kinase ROP5 of *Toxoplasma gondii* has been found to contribute to virulence and host immune evasion [65]. Also, the metacapase 4 (MCA4) of *Trypanosoma brucei* plays a role in blood stage parasite cytokinesis and virulence despite having its active site histidine altered to serine, resulting in no proteolytic activity [66].

Our finding that SMIPP-Cs are transcribed and expressed in all burrowing stages may indicate an important role of these proteins in the parasitic life style. The highest expression of SMIPP-Cs were seen in female mites (Fig. 5). Female scabies mites play a major role in transmission and establishment of the disease [4]. Similarly, only the two spotted spider mite (*Tetranychus urticae*) females emigrate and colonize, and the highest levels of digestive proteins are expressed mainly in adult stages [67]. To confirm and to determine the location of expression within the mite body we generated antibodies against representative SMIPP-Cs from three different clades within the human mite SMIPP-C protein family. Notably, the grouping of the SMIPP-Ca protein into a different clade from SMIPP-Cc and SMIPP-Ce (Fig. 2) may explain the observed lack of cross reactivity of antibodies against SMIPP-Ca to the other SMIPP-Cs (Fig. 3). Even so, all three antibodies stained solely and consistently the intestinal tract and the faeces (Fig. 4), identifying the SMIPP-Cs as secreted intestinal and excretory proteins.

Similarly, the HDM [68] and the *Psoroptes* mite [69] group one allergens have been localised in the mite digestive system and in its excretions. These potent allergens present in the mite faeces have been hypothesised to interfere with the host inflammatory response in affected tissues to trigger allergic responses [70]. The active scabies mite cysteine proteases *Sar s* 1 a-e likely have similar digestive functions, and interestingly there is also expansion of this family compared to the counterparts in free-living mites.

The mite intestinal tract and its content are certainly an important yet vulnerable compartment of the scabies parasite. While the rest of the organism is physically protected against host defence mechanisms by an impermeable chitin armour, this organ makes available a very large interface with the physiology of the host epidermis, to allow nutrient uptake and excretion. Digestion and nutrient uptake must occur through the gut lining while its damage through host defence mechanisms must be prevented. Many parasitic arthropods have evolved multiple ways to overcome this problem (reviewed in [71]). The scabies mite produces 33 gut proteins that are closely related to the HDM group 3 allergen. One of them, the serine protease *Sar s* 3, cleaves human filaggrin, thereby contributing to the breakdown of the epidermal barrier as the mite burrows within the epidermis [38]. The remaining 32 serine protease-like molecules (SMIPP-Ss [41]) contain mutations in the conserved active-site catalytic triad that are predicted to render them catalytically inactive. Some SMIPP-Ss have been shown to inhibit complement activation [40, 72] by interfering with the lectin pathway of complement activation [73], thereby protecting the mite gut lining from complement-mediated damage and enabling the mite to evade the host immune system. Two SMIPP-S crystal structures were generated [41] to elucidate the evolution of this functional change. Another class of mite proteins, namely the scabies mite SMSs, are also localised in the mite gut and in faecal pellets and also intefere with the complement cascade [42]. The accumulation of multiple mite complement inhibitors in the confined space of the mite burrow is thought to promote the survival of scabies associated pathogenic bacteria [72, 74, 75]. The fact that SMIPP-Cs are gut localised and released into the epidermis may be indicative of a role in host-parasite interaction. As shown for the SMIPP-Ss, the SMIPP-Cs may interfere with host proteins that are ingested or present in the immediate vicinity of the mite. The similarities in the evolution of these unique families emphasizes this possibility. Although the interactions of SMIPP-Cs with host mechanisms remain to be elucidated, we have delivered here the groundwork for future functional characterisation by providing evidence of when, where and in what relative quantities they are expressed throughout the parasite life-cycle.

## Conclusion

The family of the SMIPP-Cs is translated and expressed, consequently the genes with mutated catalytic diads are not redundent genes in the mite genome. Their amplification into a multicopy family and their presence within three varieties of scabies mites and expression in the mite intestinal system indicate a definite role of these proteins. Their availability to interact with host epidermal tissue and host defence systems may highlight an essential role in parasitism.

## Additional files

Additional file 1: **Table S1.** Protein and scaffold accession numbers of *S. scabiei* SMIPP-Cs and homologous HDM and scabies mite cysteine proteases. (DOCX 16 kb)

Additional file 2: **Figure S1.** Phylogenetic tree inferred from a Maximum Likelihood approach implemented in RAxML. Numbers at nodes represent bootstrap values based on 100 iterations. The tree was rooted using *Sarcoptes scabiei* var *hominis* *Sar s* 1 allergen protein sequences (Yv4003H01, Yv9053H09, Yv6030H07). (DOCX 87 kb)

Additional file 3: **Figure S2.** Western analysis of whole mite extract. Lane 1: Coomaasie blue stain. Western blots using sera raised against SMIPP-Ca (Lane 2), SMIPP-Cc (Lane 3) and SMIPP-Ce (Lane 4). (DOCX 197 kb)

## References

[1] Bornstein S, Mörner T, Samuel WM, Margo S, Pybus J, Kocan AA (2008). *Sarcoptes scabiei* and sarcoptic mange. Parasitic Diseases of Wild Mammals.

[2] Karimkhani C, Colombara DV, Drucker AM, Norton SA, Hay R, Engelman D (2017). The global burden of scabies: a cross-sectional analysis from the Global Burden of Disease Study 2015. Lancet Infect Dis.

[3] Andrews RM, McCarthy J, Carapetis JR, Currie BJ (2009). Skin disorders, including pyoderma, scabies, and tinea infections. Pediatr Clin North Am.

[4] Chosidow O (2006). Scabies. N Engl J Med.

[5] Fuller LC (2013). Epidemiology of scabies. Curr Opin Infect Dis.

[6] Engelman D, Kiang K, Chosidow O, McCarthy J, Fuller C, Lammie P (2013). Toward the global control of human scabies: introducing the International Alliance for the Control of Scabies. PLoS Negl Trop Dis.

[7] Liu X, Walton S, Mounsey K (2014). Vaccine against scabies: necessity and possibility. Parasitology.

[8] Strong M, Johnstone P (2007). Interventions for treating scabies. Cochrane Database Syst Rev.

[9] Bernigaud C, Fang F, Fischer K, Lespine A, Aho LS, Dreau D (2016). Preclinical study of single-dose moxidectin, a new oral treatment for scabies: efficacy, safety, and pharmacokinetics compared to two-dose ivermectin in a porcine model. PLoS Negl Trop Dis.

[10] Currie BJ, Harumal P, McKinnon M, Walton SF (2004). First documentation of *in vivo* and *in vitro* ivermectin resistance in *Sarcoptes scabiei*. Clin Infect Dis.

[11] Mounsey KE, Holt DC, McCarthy J, Currie BJ, Walton SF (2008). Scabies: molecular perspectives and therapeutic implications in the face of emerging drug resistance. Future Microbiol.

[12] Fischer K, Holt DC, Harumal P, Currie BJ, Walton SF, Kemp DJ (2003). Generation and characterization of cDNA clones from *Sarcoptes scabiei* var. *hominis* for an expressed sequence tag library: identification of homologues of house dust mite allergens. Am J Trop Med Hyg.

[13] Mofiz E, Holt DC, Seemann T, Currie BJ, Fischer K, Papenfuss AT (2016). Genomic resources and draft assemblies of the human and porcine varieties of scabies mites, *Sarcoptes scabiei* var. *hominis* and var. *suis*. Gigascience.

[14] Rider SD, Morgan MS, Arlian LG (2015). Draft genome of the scabies mite. Parasit Vectors.

[15] Morgan MS, Arlian LG, Rider SD, Grunwald WC, Cool DR (2016). A proteomic analysis of *Sarcoptes scabiei* (Acari: Sarcoptidae). J Med Entomol.

[16] Arlian LG, Morgan MS, Rider SD (2016). *Sarcoptes scabiei*: genomics to proteomics to biology. Parasit Vectors.

[17] Fernando DD, Marr EJ, Zakrzewski M, Reynolds SL, Burgess STG, Fischer K (2017). Gene silencing by RNA interference in *Sarcoptes scabiei*: a molecular tool to identify novel therapeutic targets. Parasit Vectors.

[18] McKerrow JH, Caffrey C, Kelly B, Loke P, Sajid M (2006). Proteases in parasitic diseases. Annu Rev Pathol.

[19] O’Brien TC, Mackey ZB, Fetter RD, Choe Y, O’Donoghue AJ, Zhou M (2008). A parasite cysteine protease is key to host protein degradation and iron acquisition. J Biol Chem.

[20] Law RH, Smooker PM, Irving JA, Piedrafita D, Ponting R, Kennedy NJ (2003). Cloning and expression of the major secreted cathepsin B-like protein from juvenile *Fasciola hepatica* and analysis of immunogenicity following liver fluke infection. Infect Immun.

[21] Barrett AJ (2004). Bioinformatics of proteases in the MEROPS database. Curr Opin Drug Discov Devel.

[22] Bhargava A, Cotton JA, Dixon BR, Gedamu L, Yates RM, Buret AG (2015). *Giardia duodenalis* surface cysteine proteases induce cleavage of the intestinal epithelial cytoskeletal protein villin *via* myosin light chain kinase. PLoS One.

[23] Olivos-Garcia A, Tello E, Nequiz-Avendano M, Gonzalez-Canto A, Lopez-Vancell R, Garcia de Leon MC (2004). Cysteine proteinase activity is required for survival of the parasite in experimental acute amoebic liver abscesses in hamsters. Parasitology.

[24] Sajid M, McKerrow JH (2002). Cysteine proteases of parasitic organisms. Mol Biochem Parasitol.

[25] Blackman MJ (2000). Proteases involved in erythrocyte invasion by the malaria parasite: function and potential as chemotherapeutic targets. Curr Drug Targets.

[26] Ikeda T (2003). Involvement of cysteine proteinases in excystment of *Paragonimus ohirai* metacercariae induced by sodium cholate and A23187. J Helminthol.

[27] Maule AG (2006). Parasitic flatworms: molecular biology, biochemistry, immunology and physiology In: Maule AG, Marks N, editors. Parasitic Flatworms: Molecular Biology, Biochemistry, Immunology and Physiology.

[28] Donnelly S, Dalton J, Robinson M. How pathogen-derived cysteine proteases modulate host immune responses. Adv Exp Med Biol. 2011;712:192–207. 10.1007/978-1-4419-8414-2_12 PMC7123607 21660666

[29] Lecaille F, Kaleta J, Brömme D (2002). Human and parasitic papain-like cysteine proteases: their role in physiology and pathology and recent developments in inhibitor design. Chem Rev.

[30] Nisbet AJ, Billingsley PF (1999). Immunological control of scab mites: digestive enzymes as candidate compounds. Vet Parasitol.

[31] Willadsen P (2006). Vaccination against ectoparasites. Parasitology.

[32] Selzer PM, Pingel S, Hsieh I, Ugele B, Chan VJ, Engel JC (1999). Cysteine protease inhibitors as chemotherapy: Lessons from a parasite target. Proc Natl Acad Sci USA.

[33] McKerrow JH (1999). Development of cysteine protease inhibitors as chemotherapy for parasitic diseases: insights on safety, target validation, and mechanism of action. Int J Parasitol.

[34] Levi A, Mumcuoglu KY, Ingber A, Enk CD (2011). Assessment of *Sarcoptes scabiei* viability *in vivo* by reflectance confocal microscopy. Lasers Med Sci.

[35] Holt DC, Burgess ST, Reynolds SL, Mahmood W, Fischer K (2013). Intestinal proteases of free-living and parasitic astigmatid mites. Cell Tissue Res.

[36] Holt DC, Fischer K (2013). Novel insights into an old disease: recent developments in scabies mite biology. Curr Opin Infect Dis.

[37] Fischer K, Holt D, Currie B, Kemp D (2012). Scabies: important clinical consequences explained by new molecular studies. Adv Parasitol.

[38] Beckham SA, Boyd SE, Reynolds S, Willis C, Johnstone M, Mika A (2009). Characterization of a serine protease homologous to house dust mite group 3 allergens from the scabies mite *Sarcoptes scabiei*. J Biol Chem.

[39] Holt DC, Fischer K, Allen GE, Wilson D, Wilson P, Slade R (2003). Mechanisms for a novel immune evasion strategy in the scabies mite *Sarcoptes scabiei*: a multigene family of inactivated serine proteases. J Invest Dermatol.

[40] Bergstrom FC, Reynolds S, Johnstone M, Pike RN, Buckle AM, Kemp DJ (2009). Scabies mite inactivated serine protease paralogs inhibit the human complement system. J Immunol.

[41] Fischer K, Langendorf CG, Irving JA, Reynolds S, Willis C, Beckham S (2009). Structural mechanisms of inactivation in scabies mite serine protease paralogues. J Mol Biol.

[42] Mika A, Reynolds SL, Mohlin FC, Willis C, Swe PM, Pickering DA (2012). Novel scabies mite serpins inhibit the three pathways of the human complement system. PLoS One.

[43] Holt DC, Fischer K, Pizzutto SJ, Currie BJ, Walton SF, Kemp DJ (2004). A multigene family of inactivated cysteine proteases in *Sarcoptes scabiei*. J Invest Dermatol.

[44] Shakib F, Gough L (2000). The proteolytic activity of Der p 1 selectively enhances IgE synthesis: a link between allergenicity and cysteine protease activity. Clin Exp Allergy.

[45] Lee AJ, Machell J, Van Den Broek AH, Nisbet AJ, Miller HR, Isaac RE (2002). Identification of an antigen from the sheep scab mite, *Psoroptes ovis*, homologous with house dust mite group I allergens. Parasite Immunol.

[46] Chapman MD, Wunschmann S, Pomes A (2007). Proteases as Th2 adjuvants. Curr Allergy Asthma Rep.

[47] Todd AE, Orengo CA, Thornton JM (2002). Sequence and structural differences between enzyme and nonenzyme homologs. Structure.

[48] Reynolds S, Fischer K (2015). Pseudoproteases: mechanisms and function. Biochem J.

[49] Sievers F, Wilm A, Dineen D, Gibson TJ, Karplus K, Li W (2011). Fast, scalable generation of high-quality protein multiple sequence alignments using Clustal Omega. Mol Syst Biol.

[50] Nielsen H (2017). Predicting secretory proteins with SignalP. Methods Mol Biol.

[51] Schultz J, Milpetz F, Bork P, Ponting CP (1998). SMART, a simple modular architecture research tool: Identification of signaling domains. Proc Natl Acad Sci USA.

[52] Gupta R, Jensen LJ, Brunak S (2002). Orphan protein function and its relation to glycosylation. Ernst Schering Res Found Workshop.

[53] Ferre F, Clote P (2005). DiANNA: a web server for disulfide connectivity prediction. Nucleic Acids Res.

[54] Combet C, Blanchet C, Geourjon C, Deleage G (2000). NPS@: network protein sequence analysis. Trends Biochem Sci.

[55] Rice P, Longden I, Bleasby A (2000). EMBOSS: the European Molecular Biology Open Software Suite. Trends Genet.

[56] Katoh K, Standley DM (2013). MAFFT multiple sequence alignment software version 7: improvements in performance and usability. Mol Biol Evol.

[57] Capella-Gutierrez S, Silla-Martinez JM, Gabaldon T (2009). trimAl: a tool for automated alignment trimming in large-scale phylogenetic analyses. Bioinformatics.

[58] Darriba D, Taboada GL, Doallo R, Posada D (2011). ProtTest 3: fast selection of best-fit models of protein evolution. Bioinformatics.

[59] Ronquist F, Huelsenbeck JP (2003). MrBayes 3. Bayesian phylogenetic inference under mixed models. Bioinformatics.

[60] Stamatakis A (2014). RAxML version 8: a tool for phylogenetic analysis and post-analysis of large phylogenies. Bioinformatics.

[61] Rapp CM, Morgan MS, Arlian LG (2006). Presence of host immunoglobulin in the gut of *Sarcoptes scabiei* (Acari: Sarcoptidae). J Med Entomol.

[62] Willis C, Fischer K, Walton SF, Currie BJ, Kemp DJ (2006). Scabies mite inactivated serine protease paralogues are present both internally in the mite gut and externally in feces. Am J Trop Med Hyg.

[63] Arlian LG (1989). Biology, host relations, and epidemiology of *Sarcoptes scabiei*. Annu Rev Entomol.

[64] Chan TF, Ji KM, Yim AK, Liu XY, Zhou JW, Li RQ (2015). The draft genome, transcriptome, and microbiome of *Dermatophagoides farinae* reveal a broad spectrum of dust mite allergens. J Allergy Clin Immunol.

[65] Reese ML, Zeiner GM, Saeij JP, Boothroyd JC, Boyle JP (2011). Polymorphic family of injected pseudokinases is paramount in *Toxoplasma* virulence. Proc Natl Acad Sci USA.

[66] Proto WR, Castanys-Munoz E, Black A, Tetley L, Moss CX, Juliano L (2011). *Trypanosoma brucei* metacaspase 4 is a pseudopeptidase and a virulence factor. J Biol Chem.

[67] Santamaría ME, Hernández-Crespo P, Ortego F, Grbic V, Grbic M, Diaz I (2012). Cysteine peptidases and their inhibitors in *Tetranychus urticae*: a comparative genomic approach. BMC Genomics.

[68] Thomas B, Heap P, Carswell F (1991). Ultrastructural localization of the allergen Der p I in the gut of the house dust mite *Dermatophagoides pteronyssinus*. Int Arch Allergy Immunol.

[69] Nisbet AJ, MacKellar A, McLean K, Brennan GP, Huntley JF (2007). Eukaryotic expression of recombinant Pso o 1, an allergen from *Psoroptes ovis*, and its localization in the mite. Parasitology.

[70] Tovey ER, Chapman MD, Platts-Mills TA (1981). Mite faeces are a major source of house dust allergens. Nature.

[71] Barros VC, Assumpção JG, Cadete AM, Santos VC, Cavalcante RR, Araújo RN (2009). The role of salivary and intestinal complement system inhibitors in the midgut protection of triatomines and mosquitoes. PLoS One.

[72] Mika A, Reynolds SL, Pickering D, McMillan D, Sriprakash KS, Kemp DJ (2012). Complement inhibitors from scabies mites promote streptococcal growth - a novel mechanism in infected epidermis?. PLoS Negl Trop Dis.

[73] Reynolds SL, Pike RN, Mika A, Blom AM, Hofmann A, Wijeyewickrema LC (2014). Scabies mite inactive serine proteases are potent inhibitors of the human complement lectin pathway. PLoS Negl Trop Dis.

[74] Swe PM, Reynolds SL, Fischer K (2014). Parasitic scabies mites and associated bacteria joining forces against host complement defense. Parasite Immunol.

[75] Swe PM, Christian LD, Lu HC, Sriprakash KS, Fischer K (2017). Complement inhibition by *Sarcoptes scabiei* protects *Streptococcus pyogenes* - an *in vitro* study to unravel the molecular mechanisms behind the poorly understood predilection of *S*. *pyogenes* to infect mite-induced skin lesions. PLoS Negl Trop Dis.

