# Supplementary material for: Phylogenetic relationships, stage-specific expression and localisation of a unique family of inactive cysteine proteases in Sarcoptes scabiei
Source: Parasit Vectors. 2018 May 16;11:301. doi: 10.1186/s13071-018-2862-0 (PMC5956821; doi:10.1186/s13071-018-2862-0)
Supplement: Supplementary file 1 — Table S1. Protein and scaffold accession numbers of S. scabiei SMIPP-Cs and homologous HDM and scabies mite cysteine proteases. (DOCX 16 kb) [file 13071_2018_2862_MOESM1_ESM.docx]

**table S1:** Protein and scaffold accession numbers of *S. scabiei* SMIPP-Cs and homologous HDM and scabies mite cysteine proteases

|  | Human mite  (*S. scabiei* var. *hominis*) | Dog mite  (*S. scabiei* var. *canis*) | Pig mite  (*S. scabiei* var. *suis)* |
| --- | --- | --- | --- |
| SMIPP-Ca | AAS93672.1 | KPM05046.1 | scaffold37186_cov165 (pig_unwashed) |
| SMIPP-Cb | AAS93673.1 | KPM11771.1 | scaffold38284_cov142 (pig_unwashed) |
| SMIPP-Cc | AAS93675.1 | KPM06027.1 | scaffold25383_cov157 (pig_washed3) |
| SMIPP-Cd | AAS93676.1 | - | - |
| SMIPP-Ce | AAS93674.1 | KPM05045.1 | scaffold3676_cov138 (pig_unwashed) |
| SMIPP-Cf | - | KPM05044.1 | scaffold446_cov160 (pig_washed3) |
| *Der p* 1 | AAB60215.1 |  |  |
| *Der f* 1 | OTF70625.1 |  |  |
| *Eur m* 1 | BAC53948.1 |  |  |
| *Sar s* 1a | AAS93667.1, |  |  |
| *Sar s* 1b | AAS93668.1, |  |  |
| *Sar s* 1c | AAS93670.1, |  |  |
| *Sar s* 1d | AAS93669.1 |  |  |
| *Sar s* 1e | AAS93671.1 |  |  |
