# Supplementary material for: Phylogenetic relationships, stage-specific expression and localisation of a unique family of inactive cysteine proteases in Sarcoptes scabiei
Source: Parasit Vectors. 2018 May 16;11:301. doi: 10.1186/s13071-018-2862-0 (PMC5956821; doi:10.1186/s13071-018-2862-0)
Supplement: Supplementary file 2 — Figure S1. Phylogenetic tree inferred from a Maximum Likelihood approach implemented in RAxML. Numbers at nodes represent bootstrap values based on 100 iterations. The tree was rooted using Sarcoptes scabiei var hominis Sar s 1 allergen protein sequences (Yv4003H01, Yv9053H09, Yv6030H07). (DOCX 87 kb) [file 13071_2018_2862_MOESM2_ESM.docx]

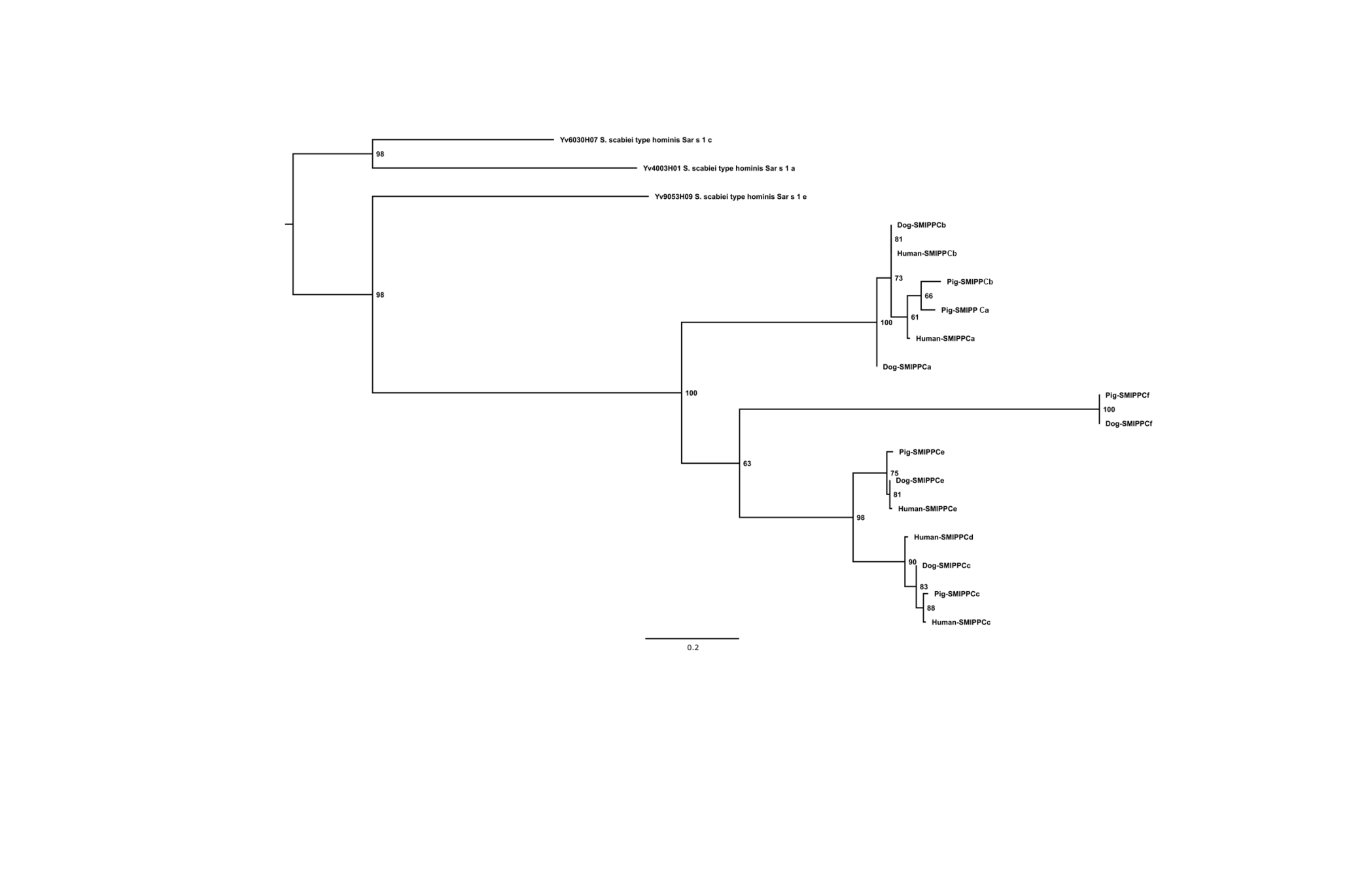


Figure S1: Phylogenetic tree inferred from a Maximum Likelihood approach implemented in RAxML. Numbers at nodes represent Bootstrap values based on 100 iterations. The tree was rooted using *Sarcoptes scabiei* var *hominis* Sar s 1 allergen protein sequences (Yv4003H01, Yv9053H09, Yv6030H07).
