# Supplementary material for: Phylogenetic relationships, stage-specific expression and localisation of a unique family of inactive cysteine proteases in Sarcoptes scabiei
Source: Parasit Vectors. 2018 May 16;11:301. doi: 10.1186/s13071-018-2862-0 (PMC5956821; doi:10.1186/s13071-018-2862-0)
Supplement: Supplementary file 3 — Figure S2. Western analysis of whole mite extract. Lane 1: Coomaasie blue stain. Western blots using sera raised against SMIPP-Ca (Lane 2), SMIPP-Cc (Lane 3) and SMIPP-Ce (Lane 4). (DOCX 197 kb) [file 13071_2018_2862_MOESM3_ESM.docx]

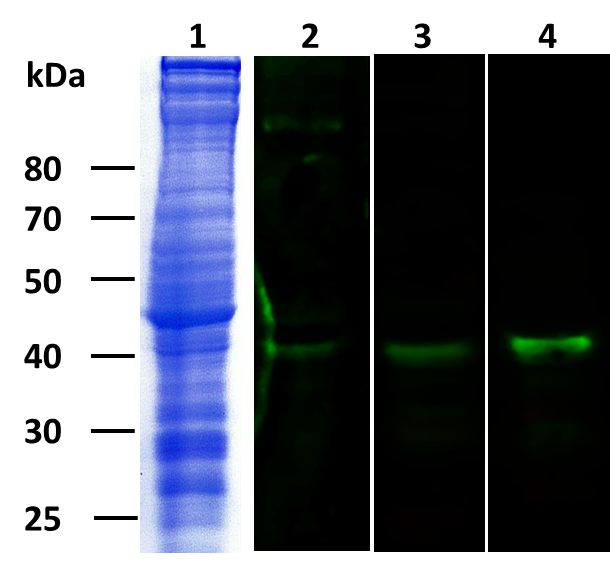


Figure S2: Western analysis of whole mite extract. Coomaasie blue stain (lane 1) and, western blots using sera raised against SMIPP-Ca (lane 2), SMIPP-Cc (lane 3) and SMIPP-Ce (lane 4).

Mixed mites (including eggs) were collected into 200ul of 2% SDS and hormogenised with a Precelly's hormogeniser at 5000rpm for 30sec at 4 degrees Celsius. The homogenised mixture was centrifuged at 12000rpm for 15min at 4 degrees Celsius. 10ul and 20ul of the supernatant was loaded for the Coomassie stained gel and for each Western blot respectively. Separate Western blots were performed using SMIPP-Ca, SMIPP-Cc and SMIPP-Ce antibodies.
